# Antiepileptic drugs and breastfeeding

**DOI:** 10.1186/1824-7288-39-50

**Published:** 2013-08-28

**Authors:** Riccardo Davanzo, Sara Dal Bo, Jenny Bua, Marco Copertino, Elisa Zanelli, Lorenza Matarazzo

**Affiliations:** 1 Division of Neonatology, Institute for Maternal and Child Health - IRCCS “Burlo Garofolo”, Via dell’Istria 65/1, Trieste 34100, Italy

**Keywords:** Antiepileptic drugs, Breastfeeding, Lactation risk

## Abstract

**Introduction:**

This review provides a synopsis for clinicians on the use of antiepileptic drugs (AEDs) in the breastfeeding mother.

**Methods:**

For each AED, we collected all retrievable data from Hale’s “Medications and Mother Milk” (2012), from the LactMed database (2013) of the National Library of Medicine, and from a MedLine Search of relevant studies in the past 10 years.

**Results:**

Older AEDs, such as carbamazepine, valproic acid, phenytoin, phenobarbital, primidone are considered to have a good level of safety during lactation, due to the long term clinical experience and the consequent amount of available data from the scientific literature. On the contrary, fewer data are available on the use of new AEDs. Therefore, gabapentin, lamotrigine, oxcarbazepine, vigabatrin, tiagabine, pregabalin, leviracetam and topiramate are compatible with breastfeeding with a less documented safety profile. Ethosuximide, zonisamide and the continue use of clonazepam and diazepam are contraindicated during breastfeeding.

**Conclusions:**

Although the current available advice on the use of AEDs during breastfeeding, given by different accredited sources, present some contradictions, most AEDs can be considered safe according to our review.

## Introduction

Breastfeeding is known for its beneficial effects on both mothers and infants [1,2]. Nevertheless, in mothers suffering from epilepsy or bipolar disorders treated with antiepileptic drugs (AEDs), some concerns on infant health may raise. The decision to encourage breastfeeding in those women should be taken after a careful evaluation of the possible side-effects on the infant caused by the indirect exposure to AEDs via breast milk. Data on the use of AEDs by the nursing woman are mainly represented by single pharmacologic or pharmacokinetic studies and/or by case reports or case series on the side-effects attributed to their presence in breast milk. Moreover, toxicological and clinical data on AEDs during breastfeeding are dispersed in the scientific literature and consequently not easily accessible to health professionals called to give an evidence-based clinical advice. The present paper aims at providing the clinician with an updated synopsis of the lactation risk of AEDs.

## Methods

Drugs considered for the present review have been selected as the most frequently used among those categorized as major antiepileptic (Code N03) by the Anatomical Therapeutic Chemical Classification System (ATC) [3] together with the anxiolytic diazepam (N05BA01). Before assessing the lactation risk of each AED, we have collected information on their main pharmacokinetic parameters: plasma protein binding, half-life, milk-to-plasma ratio, oral bioavailability (see Table 1 for definitions). We decided to present data on AEDS pharmacokinetic parameters as they represent the theoretical basis on which the lactation risk assessment should be developed. As an example, we can assume that if a drug has a short half-life (<3 hours), its level in the maternal plasma will be declining when the infant feeds again, considering a 3-hour interval. Moreover, if the drug is highly protein bound, it cannot enter the milk compartment easily. Milk/plasma ratio has the primary use of quantifying the extent of drug transfer into the milk; nevertheless, its use in assessing a lactation risk is limited as the amount of drug transfer into milk is mainly determined by the maternal plasma level. Medications for the breastfeeding mother should have a low oral bioavailability, as the result of either a poor gut absorption, or the liver sequestration prior to entering the plasma compartment. Table 2 summarises each AED main pharmacokinetic characteristics.

**Table 1:** Definitions and clinical relevance of the pharmacokinetic parameters used to assess the lactation risk following maternal intake of medications

***Pharmacokinetic parameter***	***Definition***
*Half-life or “T ½”*	The half-life of a substance is the time it takes for its plasma concentration to halve. If the half-life is long (>12-24 hrs), drugs may accumulate in maternal plasma and the time of drug transfer from plasma to breast milk is longer.
*Maternal plasma protein binding (PB)*	This parameter is expressed in percentage. The higher the percentage of the drug bound to the maternal plasma proteins, the less the drug passes into breast milk. An ideal drug to be taken during breastfeeding should have a plasma protein binding > 80%.
*Milk-to-plasma ratio (M/P)*	It denotes the ratio of the drug concentration in the mother’s milk (M) divided by its concentration in the mother’s plasma (P). It is an indicator of drug transfer into breast milk. A M/P ratio greater than 1.0 suggests that the drug may be present in breast milk in high concentrations.
*Oral bioavailability*	It describes the fraction of one orally administered dose of a drug that reaches the systemic circulation. It is expressed as a percentage of the administered dose. When the intestinal absorption of a drug is impaired, the risk of adverse effects may be lower.

**Table 2:** Main pharmacokinetic characteristics of antiepileptic drugs

	**PB**^**†**^	**Oral**	**T ½ **^**§**^	**M/P**^¶^
			**(hours)**	
		**Bioavailability **^**‡**^		
	**(%)**	**(%)**		
Carbamazepine	74	100	18 – 54	0.69
Clonazepam	50 – 86	100	18 – 50	0.33
Diazepam	99	100	43	0.2 – 2.7
Ethosuximide	NA	100	30 – 60	0.94
Gabapentin	< 3	50 – 60	5 – 7	0.7 – 1.3
Lamotrigine	55	98	29	0.057 – 1.47 [33]
Levetiracetam	< 10	100	6 – 8	0.76 – 1.55 [37,38]
Oxcarbazepine	40	100	9 (oxcarbazepine metabolyte)	0.5
Phenobarbital	51	80 – 100	20 – 133	0.4 – 0.6
			(45–500 in newborns) [41]	
Phenytoin	89	70 – 100	6 – 24	0.18 – 0.45
			(20–160 in preterm infants) [48]	
Pregabalin	NA	90	6	NA
Primidone	25	90	5 – 18	0.72
Tiagabine	96	90	7 – 9	NA
Topiramate	15	75	18 – 24	0.86 – 1.1 [55]
Valproate	94	100	14	0.42
Vigabatrin	NA	50	7	< 1
Zonisamide	40	NA	63	0.93

^†^ PB: maternal plasma protein binding expressed as percentage.

^‡^ Oral Bioavailability: intestinal absorption after oral administration expressed as percentage of the administered dose.

^§^ T ½: half-life of the drug.

^¶^ M/P: milk to plasma ratio of a drug concentration.

Data drawn from references 1–8 except where otherwise specified. NA: indicates that no data are available.

Beyond presenting pharmacokinetic parameters on AEDs, we chose to collect more relevant clinical parameters such as the theoretical infant dose (TID), the therapeutic dose in the neonatal period and the relative infant dose (RID). All these parameters were reported in a table (Table 3) together with the assessment of the lactation risk (see below), in order to have a clinical overview of each AED.

**Table 3:** Clinical relevant parameters for each antiepileptic drug and assessment of their lactation risk

**Drug**	**TID**^**†**^	**Neonatal therapeutic oral dose**	**RID**^**‡**^	**Assessment of the lactation risk according to:**		
	**(mg/kg/day)**					
			**(%)**			
				**Hale 2012**^**§**^	**LactMed 2013**	**Present study**
		**(mg/kg/day)**				
					**(including adverse drug reactions)**	
Carbamazepine	0.7	10 - 20	3.8 - 5.9	L2	▪ CBZ levels are relatively high in breast milk	Safe
					▪ Breastfed infants have serum levels that are usually below the therapeutic range.	
					▪ Side effects were rarely reported as sedation, decreased suckling, withdrawal reactions and 3 cases of liver dysfunction.	
					▪ Infant should be monitored for jaundice, drowsiness, adequate weight gain, and developmental milestones especially in premature infants, exclusively breastfed and in combination with other antipsychotics.	
Clonazepam	0.002	0.1 - 0.2	2.8	L3	▪ Monitor growth, sedation, developmental milestones, especially in preterm neonates, exclusively breastfed infants and if mother is receiving psychotropic drugs.	Contraindicated
					▪ Monitoring of serum concentration in breastfed infant, if excessive sedation occurs.	
Diazepam	0.05	IV dose available:	7.1	L3	▪ Accumulates in maternal milk and serum of breastfed infant. Other agents are preferred, especially while nursing a newborn or preterm infant.	Contraindicated
		0.1- 0.3				
		Oral dose				
					▪ Single dose does not require delaying feeding.	
		0.5 – 1 [76]				
Ethosuximide	11.5	15 – 40	31.4-73.5	L4	▪ Monitor infant for drowsiness, adequate weight gain and psychomotor development.	Contraindicated
					▪ Measurement of an infant serum level might help rule out toxicity, if there is a concern.	
Gabapentin	1.7	Only paediatric dose available:	1.3 - 6.6	L2	▪ Monitor the infant for drowsiness, adequate weight gain, and developmental milestones, especially in younger, exclusively breastfed infants and when using combinations of anticonvulsant or psychotropic drugs.	Moderately safe
		10-15				
Lamotrigine	0.7	1-6 with valproate, 5-15 with enzyme inducing AEDs	9.2	L3	▪ It is not necessary to discontinue breastfeeding, but any adverse effects such as apnoea, rash, drowsiness, decreased sucking are to be monitored and serum levels are to be measured.	Moderately safe


					▪ Monitoring of the platelet count may also be advisable.	
Levetiracetam	3.9	5 - 10 [78]	3.4 - 7.8	L3	▪ Monitor infant for the appearance of sleepiness, increase appropriate weight, normal psychomotor development.	Moderately safe
Oxcarbazepine	NA	27.7 – 50	1.5-1.7	L3	▪ Monitor the infant for drowsiness and decreased feeding, and developmental milestones especially in the first 2 months of life.	Moderately safe
		(<18 years) [72]				
Phenobarbital	0.4	3-4	24	L3	▪ The presence of phenobarbital in breast milk may mitigate possible neonatal abstinence.	Safe
					▪ Monitor the breastfed infant for the possible onset of drowsiness, adequate weight gain and developmental milestones, especially in younger, exclusively breastfed infants and antiepileptic polytherapy.	
					▪ Measurement of the infant’s serum drug concentration might help rule out toxicity.	
Phenytoin	0.4	5-8	0.6-7.7	L2	▪ The proportion ingested by infants is small and generally brings about no problems except in rare cases of idiosyncratic reactions.	Safe
Pregabalin	NA	5-14 [79]	NA	L3	▪ Compatible with breastfeeding.	Moderately Safe
					▪ An alternate drug may be preferred, especially while nursing a newborn or preterm infant.	
Primidone	0.9	12-20	8.4-8.6	L3	▪ The presence of phenobarbital in breast milk may mitigate possible neonatal abstinence.	
					▪ Monitor the breastfed infant for the possible onset of drowsiness, adequate weight gain and developmental milestones, especially in younger, exclusively breastfed infants and antiepileptic polytherapy.	
						Safe
					▪ Measurement of the infant’s serum drug concentration might help rule out toxicity.	
Tiagabine	NA	<12 years: limited data available.	NA	L3	▪ Monitor the infant for the onset of drowsiness, for adequate weight gain and for developmental milestones especially in younger, exclusively breastfed infants and when using combinations of anticonvulsant or psychotropic drugs.	Moderately safe
					▪ Other drugs should be preferred especially while nursing a newborn or preterm infant.	
Topiramate	0.3	1 - 6	24.5	L3	▪ Monitor the infant for the onset of diarrhea, drowsiness, increase appropriate weight and psychomotor development.	Moderately safe
		(<2 years) [80]				
Valproate	0.7	Limited data available in the neonatal period.	1.4-1.7	L3	▪ Breastfed infants are at risk for hepatotoxicity.	Safe
					▪ Monitor the infant for unusual bleeding (a case of thrombocytopenia has been reported).	
Vigabatrin	0.1	25-50	1.5 – 2.7	L3	▪ Until more data are available, vigabatrin should only be used with careful monitoring during breastfeeding.	Moderately safe
	*S-enantiomer*					
Zonisamide	1.9	5 – 8	28.9 -36.8	L4	▪ Monitor infant for drowsiness, adequate weight gain and psychomotor development.	Contraindicated
					▪ Measurement of an infant serum level might help rule out toxicity if there is a concern.	

^†^*TID* theoretical infant dose.

^‡^*RID* relative infant dose.

^§^ Hale lactaction risk categories: L1: safe drugs at the highest level, L2: safe, L3: moderately safe; L4: possibly dangerous, L5: contraindicated. Present study risk categories: The moderately safe category has a less documented safety profile due to a short clinical experience and lack of studies. The moderately safe AEDs can be used, but the lowest dose of the drug should be chosen and the nursing infant should be clinically monitored and, when possible, his/her plasma level should be checked.

Data drawn from reference 1,41 except where otherwise specified. NA: indicates that no data are available.

TID is the maximum estimated amount of ingested drug with breast milk; in other words, it is the estimation in milligrams per kilogram per day of the theoretical infant dose (TID). We calculated it by using the formula by Atkinson (TID = daily breast milk intake (150 ml/kg/day) × maximum breast milk concentration of medication). [4] As AEDs may be used in the neonatal period as therapy, we presented their therapeutic dose, expressed in mg/kg/day in order to have a direct comparison with the TID. A therapeutic dose which is higher than the TID reassures of the drug safety during breastfeeding. RID represents one of the most useful parameters for assessing the lactation risk. RID is calculated by dividing the infant dose via milk in “mg/kg/day” by maternal dose in “mg/kg/day”, assuming a 70 kg weight. [5] Many authors agree that anything less than 10% of the maternal dose is considered probably safe [5].

To review the lactation risk of AEDs, we consulted the following two most accredited English sources: Medications and Mother’s Milk 2012, a Manual of Lactational Pharmacology [1], and the 2013 Lactmed database (in TOXNET) [6] of the National Library of Medicine. In his textbook, updated every two years, Hale collects data on many current medications and their use during breastfeeding. After evaluating information on pharmacokinetics and what is currently published in the scientific literature for each drug, including its reported side effects, he makes a personal recommendation, using a 5 categories of lactation risk: L1: safe drugs at the highest level, L2: safe drugs, L3: moderately safe drugs, L4: drugs possibly dangerous, L5: contraindicated drugs. LactMed database is part of the National Library of Medicine’s Toxicology Data Network (TOXNET), and it includes information on the levels of drugs and other chemicals to which breastfeeding mothers may be exposed in breast milk and infant blood, together with the possible adverse effects in the nursing infant. All data published in LactMed are derived from the scientific literature and fully referenced.

To complete our synopsis, we have also performed a non-systematic Medline search of the literature with the keywords “antiepileptic drugs” AND “breastfeeding”, retrieving studies from 2004 to April 9^th^ 2013 , including the most relevant and up-dated studies on lactation risk. Data from the Committee on Drugs of the American Academy of Pediatrics (AAP) [7], and the Goodman & Gilman’s Textbook of Pharmacology [8] were also included in our review.

For each AED we organized the relevant data into small summaries.

As the result of our review we classified AEDs in 3 categories: safe, moderately safe and contraindicated during breastfeeding. The moderately safe category has a less documented safety profile due to a short clinical experience and lack of studies. However, the moderately safe AEDs can be used with caution. The lowest dose of the drug should be chosen and the nursing infant should be monitored and, when possible, his/her plasma level should be checked.

## Results

### Benzodiazepines

Benzodiazepines (BDZ) are multiple-action psychoactive compounds. Therefore, they may be used as anxiolytics, antiepileptics, sedatives, hypnotics, muscle relaxants, and as coadjuvants in anaesthesia induction. Simplifying, we can state that all BDZ produce all these effects, albeit with different expression of their principal effect (relative selectivity).

In general, the first step to judge the safety of BDZ during lactation is to know their half-life (short, intermediate, long). Independently of other kinetic characteristics, the longer is a drug half-life, the longer is its passage into maternal milk, and the greater is the infant metabolic effort to metabolize the drug. Both diazepam and clonazepam have long half-life (diazepam 43 hours, clonazepam 18–50 hours) [1]. Consequently, their primary risk to the infant is drug accumulation, which also depends on the infant metabolism (i.e. slower in preterms) and the therapy duration. Intermittent, short-term therapy (24–72 h), carries a negligible risk of accumulation, whereas prolonged use of a long half-life BDZ such as diazepam and clonazepam, carries a greater risk. In the meanwhile, there is no lactation risk after a single dose of BZD, as no drug accumulation occurs.

Diazepam is highly protein bound and its transfer into breast milk is variable. Concentrations of diazepam and its metabolite desmethyldiazepam in maternal milk vary between 7.7 and 87 ng/L and 19.2 and 77 ng/L, respectively [9]. Its active metabolite tends to accumulate during prolonged lactation. Lethargy and difficult feeding have been attributed to diazepam during breastfeeding [10]. Maternal plasma peak usually occurs within 2 h. By delaying feeding, the infant exposure to diazepam with maternal milk can be reduced.

Despite its scarce transfer into maternal milk, clonazepam was reported to cause irregular breathing, apnea and cyanosis in the first 10 days of life in an infant whose mother had been taking the drug also during pregnancy. The child psychomotor development at 5 months was normal [11]. On the contrary, in the study of Birnbaum, no side effects on breastfed infants were reported by mothers treated with benzodiazepines in association with antidepressants [12]. Clonazepam has a later plasma peak (2–6 hours) which limits the feasibility of delaying feeding in order to reduce the infant exposure.

### Carbamazepine

Carbamazepine (CBZ) is a broad-spectrum anticonvulsant, also used in psychiatric disorders (such as schizophrenia) and in trigeminal neuralgia. If taken during pregnancy, its concentration compared to the maternal serum level ranges 60-76% in the umbilical cord and 32-80% in breast milk [13]. Most available studies on carbamazepine in human milk usually refer to women under anticonvulsant polytherapy with possible interference between drugs.

The CBZ and its active epoxide metabolite (ECBZ) are poorly excreted into breast milk (M/P: 0.69 and 0.79 respectively), partly due to the good plasma protein binding. CBZ concentration in breast milk in the current literature is widely variable (0.34-6 mg/L) [14]–[17]. Its RID is low (3.8-5.9%) [1] and serum levels in babies are usually low, while ECBZ is not detected at all [18]–[20].

LactMed Database suggests to monitor breastfed infants for adequate growth and possible onset of sedation and jaundice, given some case reports of liver dysfunction with cholestasis (increase in transaminases and gammaGT) [21,22]. Nevertheless, the drug is considered compatible with breastfeeding [7,22]–[25]. In fact, it is administered, with a good safety profile, directly to patients of pediatric age.

### Ethosuximide

Ethosuximide is used in the treatment of absence seizures. Protein binding of the drug is insignificant. Its half-life is shorter in children (30–60 h) than in adults (about 45 h) and the M/P ratio is 0.94 [1]. The amount of ethosuximide excreted in breast milk leads to a relevant RID (31.4-73.5%) [1], which explains the significant serum concentrations in breastfed infants (15–40 mg/L) [26]. Although the study of Rane reported some neurological symptoms in breastfed infants (hyperexcitability, sucking difficulty), this could have been due to the antiepileptic polytherapy of their mothers and/or to the combination with withdrawal symptoms [27]. Ethosuximide is considered to be potentially dangerous during lactation [1] and monitoring breastfed infant has been suggested [26].

### Gabapentin

Gabapentin is used in the treatment of partial epilepsy, cluster headache [28], neuralgia, post-caesarean delivery pain [2] and some psychosis. Gabapentin has a medium half-life and is believed to accumulate in the fetus, while it does not concentrate in breast milk (average M/P = 0.7-1.3) [1,29], leading to low serum levels and no side effects in breastfed infants [29,30].

### Lamotrigine

In fetuses exposed to lamotrigine, concentrations are highest at birth and then gradually decline over time, more quickly if the infant is not breastfed [31]. Some of the pharmacokinetic characteristics of lamotrigine, such as the long half life (29 hours) and the low protein binding (55%) [1] together with a recent reported case of severe toxicity [32], warrant attention towards its use during breastfeeding. The anecdotal case by Nordmo refers to a 16 days-old-infant whose mother had shown visual disturbances and dizziness, after taking a high dose of lamotrigine (850 mg/day) [32]. Soon after, the baby had a series of episodes of apnea followed by a cyanotic crisis which requested resuscitation. Plasma level of lamotrigine in that infant was consistently high (4.87 μg/mL). After discontinuing the drug, the infant recovered.

The excretion of lamotrigine in breast milk is largely variable. A study on 30 breastfeeding women, treated for more than 7 days with lamotrigine at doses 300–450 mg/day, showed that breast milk samples collected over 24 h contained 0.5-18.1 μg/mL of lamotrigine [33]. The M/P ratio ranged from 0.057 to 1.47. [1,33] The dose that the breastfed infant would take has been calculated between 0.37 and 0.65 mg/kg/day, well below the dose given to infants affected by epilepsy resistant to common antiepileptic drugs [34,35]. The average RID is 9.2 (33).

Liporace reports that serum concentrations of lamotrigine in breastfed children in some cases reach therapeutic ranges [36]. These high levels may be explained by the genetic variability in the neonatal glycuroconjugation responsible for lamotrigine metabolism [36].

When maternal plasma levels are 4.5-13.4 μg/mL, breastfed infant plasma levels are 0.6-1.8 μg/mL, with a M/P ratio 0.413% on average [33]. Moreover laboratory tests (electrolytes, hepatic function tests, complete blood count) have been all normal, except from a modest thrombocytosis (range: 329.000–652.000/mm3 PLT, in 7 out of 8 infants tested) [33]. Eventually, Newport et al. reassure on lamotrigine use while breastfeeding, as no adverse effects was reported in the nursing infants in their study [33].

In conclusion, lamotrigine is considered moderately safe during breastfeeding [1,30], and LactMed recommends to check its plasma level and to carry out a platelet count in infants whose mothers are on lamotrigine [6].

### Levetiracetam

Levetiracetam is a new AED, and is usually added to other drugs in case of inadequate control of seizures. It significantly passes into breast milk (M/P: 0.76-1.55) [5,37,38], it is completely absorbed orally and it has a pediatric half-life of about 18 hours with a low RID (3.4-7.8%) [1].

Serum levels of levetiracetam in breastfed infants are low (<21 μmol/L) and no side-effects have been reported when doses between 1.3 and 3 g/day are administered to their mothers [37,38].

Consequently, levetiracetam is usually considered compatible with breastfeeding [1,37,38], even if associated to other AEDs such as primidone and phenobarbital [39].

### Oxcarbazepine

Oxcarbazepine (OXC) is a pro-drug which is converted in its active metabolite (10-OH-carbazepine) (10-OH-CBZ) in the liver. Reports of its use while breastfeeding are limited. Most information is obtained by the paper of Lutz: oxcarbazepine has a long half-life (9 h), a low M/P ratio (0.5) and a low concentration in human milk (<11 μg/mL) with a low RID (1.5-1.7%) [40]. It lacks side effects in breastfed infants. Blood levels in infants of OCX and 10-OH-CBZ are negligible (both <0.2 μg/mL). Therefore, we can consider OXC moderately safe in the breastfeeding mother.

### Phenobarbital

Phenobarbital is widely used in both adults and children. Its metabolism is mainly hepatic. The extremely long half-life in the pediatric age (20–133 hours in infants and up to 500 hours in newborns) [41] and the lower plasma protein binding in neonates compared to adults (3-43% vs 51%) could explain why its blood levels may be higher in newborns than in their mothers.

The main, yet rare, side-effect attributed to phenobarbital is sedation, holding true in adults as well as in breastfed babies. This occurrence is reported more frequently in infants whose mothers are in polytherapy, because they are likely to be exposed to interactions between drugs with possible enhancement of both clinical and side effects [39].

Phenobarbital is commonly and safely used at a daily dosage 5–7 mg/kg/day in newborns affected by seizures or drug-abstinence syndrome. This dose is far higher than the estimated dosage (2–4 mg/day) received by a breastfed infant by a woman taking 150 mg (a high dose) phenobarbital per day. Given these data, there is no need to discontinue breastfeeding in mothers treated with phenobarbital. However, AAP is more cautious and recommends to monitor all breastfed infants whose mothers are on phenobarbital [7]. In premature babies or in infants with drowsiness, difficulty in sucking or poor weight gain, it is recommended to monitor its plasma levels [6,42]–[44].

### Phenytoin

Despite the formerly reported methemoglobinemia in infants [45], phenytoin is considered to be safe in lactation [5,46,47], as it is highly bound to plasma proteins (89%) [1], it scarcely passes into breast milk (M/P: 0.18-0.45) [1,48,49] and has low concentration in human milk [49]. In fact, milk concentration of phenytoin has been reported to be only 1.9 mg/L following maternal intakes equal to 300–600 mg [50]. The amount ingested by nursing infants is usually low (RID 0.6-7.7%) [1].

### Pregabalin

Pregabalin is used to treat postoperative [51] and neuropathic pain and some psychosis. There are no studies on the passage of pregabalin into human milk. The absence of binding to plasma proteins and its excellent oral bioavailability [52] suggest that it can pass into the mother’s milk and into the circulation of the nursing infants. Numerous side effects such as dizziness, drowsiness, impaired vision have been observed in adults. It is rated as moderately safe during breastfeeding [1].

### Primidone

Primidone is metabolized to phenobarbital and other derivates. Serum levels of primidone and its metabolites in breastfed infants could be close to the therapeutic range values and cases of sedation and poor feeding are reported in literature. It should be used with caution during breastfeeding [14,42,43,53,54], also according to the AAP [7]. The clinical assessment of its use during breastfeeding is similar to phenobarbital.

### Tiagabine

Tiagabine oral absorption is almost complete and it is highly bound to plasma proteins [41]. There are no studies on its use during breastfeeding, leading to possibly prefer other antiepileptic drugs. If taken by the nursing mother, infant should be monitored [46].

### Topiramate

Topiramate use is increasingly prescribed, being effective and well-tolerated by epileptic patients. It is rapidly absorbed, it has a low plasma protein binding, a relatively long half-life and a significant excretion into breast milk (M/P:0.86-1.1) [1,55] with a high RID (24.5%) [1]. Despite these characteristics may raise some concerns on its use during breastfeeding, breastfed infants have been found to have very low serum levels (<2.8 μmol/L) in the first 3 months of life after maternal intakes of 150–200 mg/day of topiramate in association with CBZ [55]. These low serum levels seem to depend on the infant good capability to eliminate topiramate, possibly facilitated by CBZ enzyme induction [55]. Moreover, infants who were breastfed by mothers treated with topiramate did not show side-effects. Notwithstanding, they should be monitored [55,56].

### Valproate

Patients taking valproate may develop hepatotoxicity, thrombocytopenia and anemia. The limited passage of valproate into breast milk (the drug is almost completely bound to plasma proteins) make it safe in lactation. [23,24,46,47] Serum levels in breastfed infants are low [18]. However, some controversies exist on its safety profile. The teratogenic effect of valproate exposure in pregnancy [57], together with the decrease of methylation in the DNA extracted from the umbilical cord blood [58] and a report of a breastfed infant with thrombocytopenia, purpura and anemia [59], induce a cautious use of this AED during breastfeeding [1]. Although there is no indication to perform routine laboratory investigations, in case of late jaundice, it is reasonable to assess the hepatobiliary function and to test its plasma levels in the breastfed infant [23,46,57]–[62].

### Vigabatrin

Vigabatrin is commonly used for multi-resistant epilepsy. The maternal plasma concentration is 93 μmol/L following an intake of 1.5 g vigabatrin. There are no precise data about its passage into breast milk. Since no information is available about its use during lactation, the nursing infants should be monitored [46].

### Zonisamide

Zonisamide is a broad-spectrum anticonvulsant with a half-life of nearly 63 hours [1], even if some authors report a longer one (109 hours) [63]. It easily passes into breast milk (M/P: 0.93) [6,64]. Although no studies document side-effects in children of mothers receiving zonisamide, due to a very high RID (28.9-36.8%), it has been considered contraindicated by some authors [1]. Others authors simply recommend measurement of infant serum level to rule out toxicity, if there is a concern [6].

Table 2 summarises each AED main pharmacokinetic characteristics. Table 3 gives an overview of the clinical relevant parameters for each antiepileptic drug (TID, therapeutic dose, RID) and the assessment of their lactation risk according to the two main sources (Hale and LactMed) and to our study group.

## Discussion

The use of AEDs is often unavoidable for most mothers with epilepsy. Moreover, the post partum period is a vulnerable time for women with epilepsy, owing to the changes in the drug metabolism and possibly to sleep deprivation. Both these conditions may trigger an epileptic crisis [65].

As the newborn might be indirectly exposed to AEDs via breast milk, pharmacologically treated epilepsy has been and sometimes is still considered a contraindication to breastfeeding, irrespective of which AED is taken by the mother [66]. On the contrary, health professionals have currently become more aware of the need to weigh the short and long-term risks for the nursing infant against the well demonstrated nutritional, immunological, developmental, economic and ecological benefits of breastfeeding [1,2,67]. When evaluating the safety profile of an AED during lactation, the physician should collect and process data which are often dispersed in the scientific literature and difficult to retrieve [66]. We have therefore completed the present review in order to facilitate the consultancy on the use of AEDs during breastfeeding.

The comparison of advices from the main and most recent accredited literature sources has highlighted the existence of a certain degree of inconsistency [66], which is probably the consequence of the combination of limited scientific evidence and of a certain degree of arbitrariness. This heterogeneity of advice is not specific of AEDs. We have previously documented a metavariability in the assessment of the lactation risk of other classes of drugs, such as beta-blockers [68], corticosteroids [69] and antidepressants [70].

Evaluating the lactation risk of any drug is complex and should take into account several aspects. In our review, we have shown that neither pharmacokinetic information nor more clinical parameters (such as TID and RID) are alone good predictors of the lactation risk. As an example, a drug such as phenobarbital, which is poorly bound to the mother plasma proteins and has a relatively long half-life and an high RID, can still be considered compatible during breastfeeding [1].

When evaluating the lactation risk of a drug, we should always check whether or not toxicity in breastfed infants is reported. AEDs are expected to determine in the nursing infant a series of symptoms related to their pharmacological effects on the central nervous system, such as altered sleep patterns, abnormal tone, poor feeding and possibly poor growth secondary to inadequate breast sucking. However, the clinical relevance of these attributed side-effects vary widely. As an example, the lactation risk of infant apnea after maternal use of clonazepam [11] deserves a higher level of caution than the risk of infant sedation after maternal use of CBZ.

Another variable to take into account when considering the infant tolerance to AEDs, is his/her ability to metabolize the drug. This is much lower when the maternal treatment begins before or during pregnancy (as the exposure of the baby through breast milk adds to the one through the placenta), or in case of prematurity and during the first 2 months of life [71]. Maternal polytherapy is also expected to increase the lactation risk, as the result of the possible synergistic interaction of different medications [39].

We might also speculate that the alleged long-term negative effects on child psychomotor development resulting from the passage of small amounts of the drug through breast milk, though up to now not well-documented, could be well-balanced by the benefits that breastfeeding confers to the cognitive, social and relational development. This hypothesis seems to be supported by the results from a study conducted in the US on 199 children exposed in uterus to AEDs (phenytoin, carbamazepine, valproate and lamotrigine) and followed in the first 3 years of life. In this cohort, there was no significant difference regarding cognitive scores between breastfed and not breastfed children [24].

Breastfeeding mothers should be provided sound and clear information on the lactation risk of the prescribed medications. Nevertheless, breastfeeding mothers taking AEDs happen to receive inconsistent and sometimes conflicting advices on whether or not to breastfeed from different clinicians (general physician, neurologist, pediatrician, obstetrician, etc.). Unluckily, many such given advices are not evidence-based, but simply emphasize not circumstantial assessments of the lactation risk.

We believe that mothers treated with AEDs should be encouraged to start breastfeeding immediately after childbirth, even if a definite and complete evidence-based information has yet not been collected. In fact, breast milk should not be considered as a “presumed guilty”, because, after a complete scrutiny, only very few AEDs resulted contraindicated during breastfeeding [24,72,73].

The present study is not without limitations. First of all, we did not conduct a systematic review of the published literature, but we offered a synopsis of the two most updated and authoritative clinical sources on the lactation risk (namely: Hale and Toxnet). Secondly, the existing reports on the lactation risks of medications in general [73], and particularly of AEDs [44] , are usually anecdotal or based on case series and therefore of poor methodological quality. However, we should remember that any recorded side-effect, while biologically and pharmacologically plausible in the nursing infant, is rarely attributable with certainty to a particular drug taken by her/his mother [74].

## Conclusions

According to the present review, we grouped AEDs into 3 main classes of lactation risk:

1. *AEDs safe to use during lactation*. Carbamazepine, valproic acid and phenytoin are compatible with breastfeeding. Phenobarbital and primidone are also compatible, although particular attention should be paid on infant monitoring, possibly measuring the infant plasma drug level.

2. *AEDs with a less documented safety profile during lactation (moderately safe)*. Gabapentin, lamotrigine, levetiracetam, oxcarbazepine, pregabalin and vigabatrin may be used during lactation, but their lowest dose should be prescribed. The nursing infant should be carefully monitored and, if required, the infant drug plasma-level tested. When lamotrigine is taken by the mother, it is advisable to check the presence of thrombocytopenia in the nursing infant. Although based on scarce available data, tiagabine and topiramate can be considered compatible with breastfeeding.

3. *AEDs contraindicated during lactation*. Ethosuximide should be used only when there are no alternatives and after informed consent of the mother. Lastly, zonisamide and the continuous use of diazepam or clonazepam should be avoided.

Even if an AED can be safely taken by the breastfeeding mother, it is a good clinical practice to call the mother attention on the behavior, sleep, feeding patterns and growth of the nursing infant, especially in the first 2 months of life [75]. When possible, we might advice to monitor the infant plasma levels, especially for the moderately safe AEDs. However, we are aware that there is no clear agreement on when to test them. We believe that after 4–8 weeks is a reasonable time window, as, if the child is fully breastfed, breast milk would have reached its highest production and the consequent intake by the nursing infant would be at the maximum.

## Competing interests

This study was performed without any funding or grants. All the authors declare that they have no competing interest and do not have any financial relationships with any biotechnology and/or pharmaceutical manufacturers.

## Authors’ contributions

RD conception and design. LM, SDB, RD analysis and interpretation of the data. LM, SDB RD drafting of the article. RD critical revision of the article for important intellectual content. All authors listed here have seen and approved the final version of the report. JB administrative, technical, or logistic support. MC, EZ collection and assembly of data.
